# Previous beliefs affect Bayesian reasoning in conditions fostering gist comprehension

**DOI:** 10.3758/s13421-023-01435-1

**Published:** 2023-06-02

**Authors:** Elisabet Tubau, Àngels Colomé, Javier Rodríguez-Ferreiro

**Affiliations:** Department of Cognition, Development and Educational Psychology Institute of Neurosciences University of Barcelona, Pg Vall d’Hebron, 171, O8035, Barcelona, Spain

**Keywords:** Bayesian reasoning, Belief effect, Presentation format, Nonnumerical probability estimates, Gist comprehension

## Abstract

It has been shown that Bayesian reasoning is affected by the believability of the data, but it is unknown which conditions could potentiate or reduce such belief effect. Here, we tested the hypothesis that the belief effect would mainly be observed in conditions fostering a gist comprehension of the data. Accordingly, we expected to observe a significant belief effect in iconic rather than in textual presentations and, in general, when nonnumerical estimates were requested. The results of three studies showed more accurate Bayesian estimates, either expressed numerically or nonnumerically, for icons than for text descriptions of natural frequencies. Moreover, in line with our expectations, nonnumerical estimates were, in general, more accurate for believable rather than for unbelievable scenarios. In contrast, the belief effect on the accuracy of the numerical estimates depended on the format and on the complexity of the calculation. The present findings also showed that single-event posterior probability estimates based on described frequencies were more accurate when expressed nonnumerically rather than numerically, opening new avenues for the development of interventions to improve Bayesian reasoning.

**Supplementary Information:**

The online version contains supplementary material available at 10.3758/s13421-023-01435-1.

## Introduction

Bayesian reasoning tasks are generally presented as mathematical problems that request the posterior probability of a hypothesis if certain condition were true (e.g., probability that an individual who sleeps with the window open would catch a cold), based on the prior probability of that hypothesis (probability of catching a cold), and on the likelihood of the condition (likelihood of sleeping with the window open for individuals with and without a cold; see examples in the Appendix). Accordingly, correct Bayesian problem-solving implies understanding the extent to which the condition changes the prior probability (i.e., the impact of the condition; Tentori et al., 2016). However, accurate estimates of the posterior probability are rare and strong effort has been put to investigate how to improve performance (e.g., Mandel & Navarrete, 2015). In essence, besides the cognitive abilities of the reasoner and/or experience with the task, both the format of the numerical information and the believability of the scenario modulate the accuracy of the posterior probability estimates.

### Format of the data and levels of understanding

A large amount of evidence shows that presenting the data as ratios of natural frequencies makes the Bayesian inference easier, compared with percentages or decimals (see, for example, the reviews of Brase & Hill, 2015; Johnson & Tubau, 2015; McDowell & Jacobs, 2017). The benefit of the frequency format has been attributed to its similarity to the way in which the mind intuitively encodes the frequency of events in every-day tasks (e.g., Cosmides & Tooby, 1996; Gigerenzer & Hoffrage, 1995), or to its role in enhancing the comprehension of the nested-sets structure of the data (e.g., Barbey & Sloman, 2007; Girotto & Gonzalez, 2001). Nevertheless, natural frequencies described in the text do not eliminate all the difficulties of the Bayesian task and, in some conditions, they still lead to erroneous estimates that suggest superficial or incomplete understanding.

**Fig. 1:**
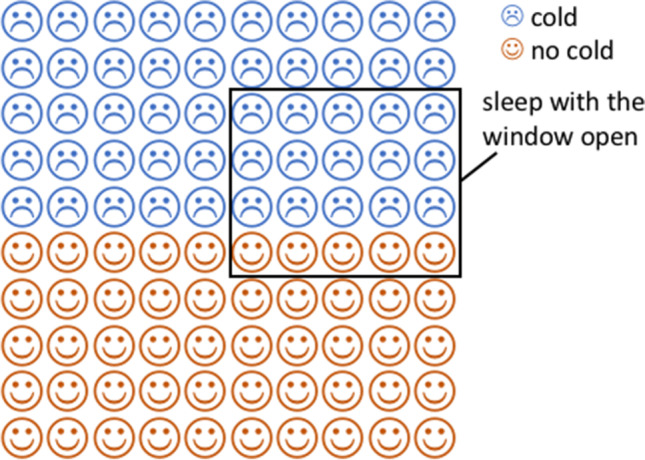
Example of icon array used in the Study 1 (see Table 1 for a description of these data)

It has been proposed that numerical data can be understood in a verbatim level (by representing the literal words and numbers and performing exact calculations) or in a more abstract level (by representing their gist or general meaning; Reyna, 2008; Furlan et al., 2016). In the Bayesian task, different verbatim levels could be distinguished. One level, based on correct calculation, would lead to a correct posterior probability estimate. The other, based on incomplete processing of the data, could lead to highly incorrect responses. As outlined above, the latter can be the case for word problems describing natural frequencies as well. For example, in Tubau et al. (2019), natural frequencies presented in text format were not better than percentages in helping to infer the posterior ratio as a single-event probability,^1^ and most of the responses suggested a superficial and incomplete understanding of the data (common errors were numbers presented in the text such as the hit rate or the base rate; see also Evans et al., 2000; Johnson & Tubau, 2017; or Pennycook & Thompson, 2012, for similar errors). Interestingly, Tubau et al. (2019) also showed that the presentation format of the frequency information modulates the level of understanding. Specifically, in contrast to the text format, where the few correct estimates were expressed using the literal numbers, an iconic display (see an example in Fig. 1) promoted mostly accurate single-event probability estimates and a comprehension of the posterior ratio beyond the literal data (about half of the participants used equivalent expressions for representing the posterior ratio, such as “1 of 5” or “20 of 100” instead of “3 of 15”).

Therefore, previous findings suggest three levels of understanding of the data included in Bayesian scenarios: verbatim-incorrect (superficial and incomplete data processing), verbatim-correct (correct exact calculation), and comprehension of the gist of the posterior probability (i.e., understanding if it is larger or smaller than the prior and the approximate distance between different probabilities). Although most exact correct Bayesian responses imply a comprehension of the gist, some of them do not. That is, in some conditions, the components of the posterior ratio can be induced by directly matching common text-question keywords, as shown in the case of the direct translation strategy in mathematical problem solving (Lewis & Mayer, 1987). This is more likely to be observed in the case of text formats and questions that guide the selection of corresponding numbers, such us two-step frequency questions (e.g., Girotto & Gonzalez, 2001; Tubau, 2022). At the same time, comprehension of the gist of the posterior ratio does not imply necessarily an exact correct response, as shown for nonnumerical representations of proportions (Ahl et al., 1992; Dixon & Moore, 1996; see also below). Importantly, these different levels may also differ in their sensitivity to contextual information such as the believability of the scenario.

### Gist comprehension and the influence of previous beliefs

Gist comprehension of numerical data seems to automatically engage the evaluation of some properties, such as its *goodness*. For example, research on decision-making suggests that affective evaluations of the prospects (i.e., whether they are good or bad; Peters et al., 2006; Petrova et al., 2019) or the goodness of medical treatments (Reyna, 2008) are based on the gist. In Bayesian scenarios, the evaluation of the reliability or believability of the data, and associated affective reactions, might also depend on the gist of the posterior. If this were the case, we expected that gist-based reasoning would be more sensitive to the believability of the data than would more verbatim processing.

Interestingly, the influence of previous beliefs in Bayesian reasoning has been reported for conditions that may foster a gist understanding of the data. For example, Evans et al. (2002) presented problems that included only one piece of information (either the prior probability of the hypothesis or the likelihood of the datum), while the other was implicit. Overall, their findings showed that the implicit information, based on participants’ beliefs, had a stronger effect on the posterior probability estimate than the explicit information. More directly related with the believability of the data, Cohen et al. (2017) showed that posterior probability estimates were more accurate for believable rather than for unbelievable scenarios. While estimates based on believable scenarios did not differ from the ones produced by a group of participants who did not see the data, unbelievable scenarios led to largely erroneous estimates. Of note, in these previous studies, participants provided approximate estimates of the posterior probabilities (by either marking a point on a line or selecting the response among radio buttons), which might trigger the comprehension of the gist to a larger extent than the request of an exact estimate.

To more directly study the relationship between gist understanding and the influence of previous beliefs, in the present studies we compared conditions that may foster this level of comprehension (nonnumerical representations of either the data, or the response) with conditions that may foster more verbatim-based processing (text descriptions of numerical data and/or requests of exact numerical estimates).

### Nonnumerical representations of proportions

Differing from ratios expressed with natural numbers, non-symbolic ratios, as the ones represented in icon arrays or in continuous lines, foster “perceptual intuitions”; that is, the intuitive comprehension of part-to-whole or part-to-part relations that adults share with infants and other nonhuman animals (Matthews & Ellis, 2018). In some conditions, intuitive understanding of non-numeric ratios may be more accurate than the comprehension of corresponding numerical ones (Matthews & Ellis, 2018). This is coherent with the observed benefit of icons, compared with numerical formats, for Bayesian reasoning (e.g., Brase, 2009, 2014; Galesic et al., 2009; Garcia-Retamero & Hoffrage, 2013; Garcia-Retamero et al., 2015; Sirota et al., 2014, Exp 2; Tubau et al., 2019). This benefit has been related to the proposal that icons facilitate proportional reasoning through the intuitive visualization of the gist of the ratio (Brust-Renck et al., 2013; Reyna, 2008; Stone et al., 2018), being considered closer to the type of information to which the human minds have been adapted (Brase, 2009, 2014). Hence, we expected to replicate the observed benefit of icons on the accuracy of posterior probability estimates. Moreover, by enhancing a gist comprehension of the data, we also expected to observe a stronger influence of previous beliefs in estimates based on icons than in those based on textual presentations.

Similarly, previous evidence suggests that nonnumerical estimates, such as the ones expressed as marks on continuous lines, are useful to either assess or foster gist comprehension of proportions in mathematical problem solving (Ahl et al., 1992; Dixon & Moore, 1996). Specifically, in the Bayesian task with text presentations, the requirement to make a single mark on a line might promote the integration of the different pieces of numerical information and, hence, fewer errors due to superficial processing would be expected. Therefore, in both iconic and text formats, the requests of nonnumerical estimates would foster a gist understanding of the data and, consequently, a stronger influence of previous beliefs than the requests of numerical estimates. These hypotheses were tested in the following studies.

### Study 1

We created two Bayesian problems related to conditions commonly considered to prevent or facilitate catching a cold. For half of the participants the data were believable, while for the other half they were unbelievable. Data were presented either as ratios of natural frequencies described in the text or as icon arrays, and participants were required to estimate the posterior probability both nonnumerically and numerically. As outlined above, we expected to observe a significant belief effect for nonnumerical estimates in either format. We also expected to observe stronger influence of previous beliefs on numerical estimates based on icons, compared with those based on natural frequencies presented in text. We requested single-event probabilities (percentages), both nonnumerically (in a continuous line) and numerically (as a percentage). It has been observed that Bayesian inferences based on described frequencies are much less accurate when they are requested as single-event probabilities rather than as frequencies in set and subset (Cosmides & Tooby, 1996; Tubau et al., 2019). This effect has been attributed to a difficulty in understanding the concept of single-event probability (Cosmides & Tooby, 1996). Nevertheless, Tubau et al. (2019) showed that single-event probability estimates based on text formats were mostly correct when the required set-subset relation aligned with the described ones (differing from the Bayesian inference, the critical numbers had the same subset role in both the description and the response^2^). By contrast, single-event probability estimates based on iconic format were mostly correct, regardless the type of question (Tubau et al., 2019). The dependency of the text format on the question-text alignment points to the involvement of superficial problem-solving strategies (i.e., direct translation; Lewis & Mayer, 1987). However, if the request of a nonnumerical estimate diminished the reliance on superficial processing, it could increase the accuracy of the probability estimates based on text presentations as well.

**Table 1:** Problems presented in the text format (natural frequency) in Study 1

	Believable	Unbelievable
**Posterior 25**	According to a recent study, 50 out of every 100 people catch a cold in winter. Of the people who catch a cold, 10 eat oranges every day. Of the 50 people who do not catch a cold, 30 also eat oranges every day.	According to a recent study, 50 out of every 100 people catch a cold in winter. Of the people who catch a cold 10 sleep with the window open. Of the 50 people who do not catch a cold, 30 also sleep with the window open.
**Posterior 75**	According to a recent study, 50 out of every 100 people catch a cold in winter. Of the people who catch a cold, 15 sleep with the window open. Of the 50 people who do not catch a cold, 5 also sleep with the window open.	According to a recent study, 50 out of every 100 people catch a cold in winter. Of the people who catch a cold, 15 eat oranges every day. Of the 50 people who do not catch a cold, 5 also eat oranges every day.
**Nonnumerical estimate request**	Based on these data, to what extent will a person who / eats oranges every day/sleeps with the window open/catch a cold?	
**Numerical estimate request**	More concretely, according to the data presented above, what is the probability that a person who / eats oranges every day / sleeps with the window open / will actually catch a cold? (%)	

Participants received both posterior 25 and 75 scenarios, being the data were either believable or unbelievable (see the appendix for an example in the iconic format).

## Method

### Participants and design

Three hundred forty-three undergraduates (mean age = 22.5 years; 47 males) participated in the experiment. One hundred seventy-nine received the data as ratios of natural frequencies described in a text (NF; see Table 1). The rest (164) received the same data through icon arrays (IA; see an example in Fig. 1). In each format, some participants received believable data (93 in NF; 78 in IA) and for the rest, the data were unbelievable (86 in NF; 86 in IA). Participants were randomly assigned to one of these groups.

### Questionnaire and procedure

Problems were presented through an online questionnaire (Qualtrics XM). First, we assessed previous beliefs by requesting participants to estimate (1) the prior probability of catching a cold for people at their age, and (2) two conditional probabilities: (2a) the probability of catching a cold if sleeping with the window open and (2b) the probability of catching a cold if eating oranges every day. Estimates had to be reported by placing the cursor on a line with two endpoints “definitely not” (on the left), and “totally sure” (on the right) and a middle point (“as likely as not”). The position of their marks was numerically coded from 0 to 100, but numbers were not displayed.

Second, two Bayesian problems related to the conditions assessed in the previous beliefs’ requests were presented to each participant in the same format (icons or natural frequencies in text format). The posterior probabilities were 25% for one problem and 75% for the other to obtain symmetrical preventive and generative conditions with respect to the prior (always 50%). However, numbers and scenarios were crossed so that, for half of the participants, the scenarios were believable while, for the other half, they were unbelievable (see Table 1). For each problem, participants were requested to estimate the corresponding posterior probability both in the line and numerically, as a percentage. Questions were the same for both iconic and text presentations (see an example of problem in the iconic format in the Appendix). The order of presentation of the problems was counterbalanced. Participants were also assessed on other cognitive abilities, as a part of a larger project that will not be discussed here.

### Ethics statement

The procedure was approved by the University of Barcelona’s Bioethics Commission. Participants were free to join in the experiment and provided written consent for the use of their data for research purposes. They were debriefed in a subsequent session.

### Data analyses

First, participants who reported inconsistent previous beliefs with our believable scenarios (P(cold|window open) <= P(cold) and P(cold|orange everyday) >= P(cold)) were eliminated from the analyses. Second, to study the effects of the different independent variables on the accuracy of the posterior probability proposed to each problem, we fitted binomial generalized linear mixed effects models (using the R lme4 package), with posterior (25 or 75) as the within-participant variable, and Format and Believability as the between-participants factors, with the participants as random effects. Two measures of accuracy were considered: exact correct (plus an estimate error for nonnumerical estimates) and approximate correct (see below). Finally, we used factorial ANOVAS to study the effects of the abovementioned factors on the distance between the estimates proposed to each problem, and chi-squared and *t* tests to study within-participants differences between nonnumerical and numerical estimates.

## Results

### Previous beliefs

Only four participants in each format condition showed inconsistent beliefs and were eliminated from the analyses. For the belief-consistent participants, the means of the estimates were 55% for P(cold), 79% for P(cold|window open) and 42% for P(cold|orange everyday). Differences between these means were all significant (*p*s < .001).

### Nonnumerical posterior probability estimates

**Fig. 2:**
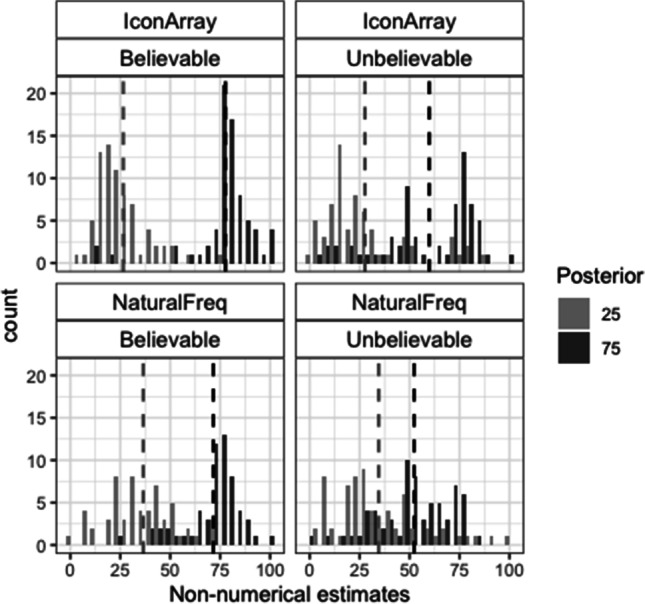
Histograms of the nonnumerical estimates in Study 1. Dashed vertical lines represent the means

Figure 2 shows the histograms of the numbers corresponding to the nonnumerical estimates proposed to each problem. As introduced, these data were analyzed based on two accuracy criteria: (1) the percentage of line marks in the actual posterior ±3 interval (this corresponds to the average estimate error for the 0–100 line observed in a sample of the same population (Núñez-Peña et al., 2019), and (2) the percentage of line marks in the correct direction (estimate of posterior 25 < 40 and estimate of posterior 75 > 60). This second criterion was created as a measure of intuitive comprehension of the relation between the prior (always 50%) and the posterior. Regarding the first criterion, the best model showed a single significant effect of posterior (loglikelihood ratio with and without this factor: −325/−328; AIC ratio: 656/660; χ^2^(1) = 6.07, *p* = .014; see Table S1-A). The percentage of estimates in the actual posterior ±3 interval was higher for the posterior 25 than for the posterior 75 (23% vs. 16%; see also Table 2-A). Regarding the second criterion, the best model revealed that all the main effects and two interactions: Posterior × Format and Posterior × Believability were significant (loglikelihood ratio between models with and without interactive effects: −396/−400, AIC: 805/810; χ^2^(2) = 8.39, *p* = .015; see Table S1-B). There were more nonnumerical estimates in the correct direction for IA than for NF (74% vs. 51%), for believable rather than unbelievable scenarios (this difference was significant only for the posterior 75: 63% vs. 41% for believable vs. unbelievable scenarios) and, in general, for the posterior 25 rather than for the posterior 75 (this difference being larger for NF: 65% vs. 37% than for IA: 79% vs. 69%; see also Table 2-B).

**Table 2:** Percentages of correct nonnumerical estimates in Study 1 (frequencies are shown inside parentheses)

	Believable		Unbelievable	
A. Actual posterior ±3	25	75	25	75
Icon Array	28 (22 of 78)	17 (13 of 78)	23 (19 of 82)	20 (16 of 82)
Natural Freq.	18 (16 of 91)	15 (14 of 91)	24 (20 of 84)	12 (10 of 84)
B. Correct direct.	25	75	25	75
Icon Array	81 (63 of 78)	81 (63 of 78)	78 (47 of 82)	57 (36 of 82)
Natural Freq.	70 (64 of 91)	47 (43 of 91)	60 (50 of 84)	26 (22 of 84)

### Numerical posterior probability estimates

**Fig. 3:**
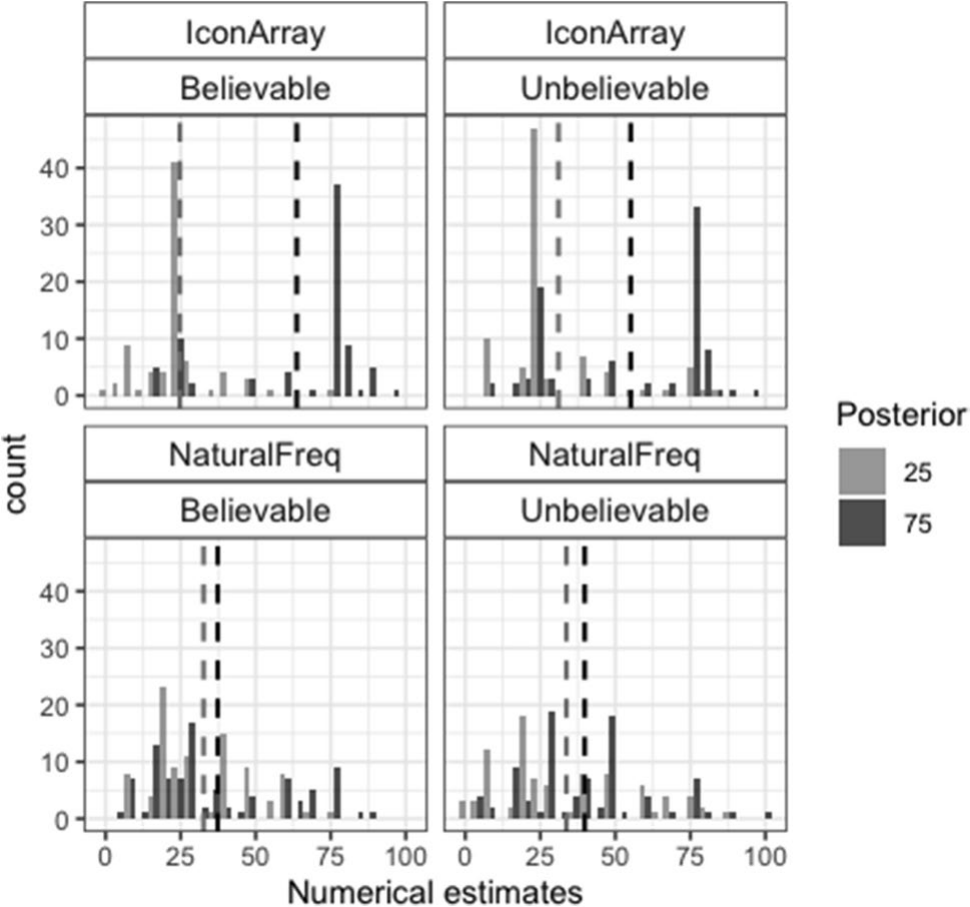
Histograms of the numerical estimates in Study 1. Dashed vertical lines represent the means

Figure 3 shows the histograms corresponding to the numerical estimates. These data were also analyzed based on two accuracy criteria: (1) percentage of exact correct responses (either 25 or 75) and (2) percentage of estimates in the correct direction. For the first criterion, the best model showed significant effects of posterior, format and a marginal interaction of these factors (loglikelihood ratio between models with and without interaction −291/−293; AIC ratio: 592/593; χ^2^(1) = 3.40, *p* = .065; see Table S2-A). There were more exact correct estimates for IA than for NF (48% vs. 9%), and for the posterior 25 than for the posterior 75 in the case of IA (53% vs. 42%; for NF this percentage was 9% in each posterior; see also Table 3-A). For the second criterion, the best model showed that all the main effects and the interaction Posterior × Format were significant (loglikelihood ratio between models with and without the interaction: −376/−379; AIC ratio: 763/768; χ^2^(1) = 6.99, *p* = .008; see Table S2-B). There were more numerical estimates in the correct direction for IA than for NF (71% vs. 38%), for believable rather than for unbelievable scenarios (59% vs. 49%), and for the posterior 25 than for the posterior 75 (this difference being larger for NF: 60% vs. 17% than for IA: 82% vs. 61%; see also Table 3-B).

**Table 3:** Percentages of correct numerical estimates in Study 1 (frequencies are shown inside parentheses)

	Believable		Unbelievable	
A. Exact correct	25	75	25	75
Icon Array	53 (41 of 78)	47 (37 of 78)	54 (44 of 82)	38 (31 of 82)
Natural Freq.	9 (8 of 91)	10 (9 of 91)	8 (7 of 84)	8 (7 of 84)
B. Correct direct.	25	75	25	75
Icon Array	88 (69 of 78)	69 (54 of 78)	76 (62 of 82)	52 (43 of 82)
Natural Freq.	60 (55 of 91)	21 (19 of 91)	60 (50 of 84)	13 (11 of 84)

### Distances between estimates

To assess the accuracy of the perception of the distance between both posterior probabilities, we analyzed the extent to which the distance between the estimates corresponded to the actual distance (50). To this end, we calculated, for each participant and for each type of estimate, a distance score (posterior 75 estimate − posterior 25 estimate). For the distances between the nonnumerical estimates, the factorial ANOVA showed significant effects of format, *F*(1, 333) = 48.04, *p* < .001, η^2^ =.12, and believability, *F*(1, 333) = 19.88, *p* < .001, η^2^ =.06. Distances were closer to the actual one for IA than for NF (means: 39 vs. 15), and for believable rather than for unbelievable data (means 34 vs. 19). For the distances between the numerical estimates, the factorial ANOVA also showed significant effects of format, *F*(1, 333) = 71.69, *p* < .001, η^2^ = .17, and believability, *F*(1, 333) = 4.42, *p* = .036, η^2^ = .01. Moreover, the Format × Believability interaction was significant, *F*(1, 333) = 7.14, *p* = .008, η^2^ = .02. Believability was significant for IA (means: 39 vs. 23 for believable vs. unbelievable scenarios), *F*(1, 158) = 11.41, *p* = .001, η^2^ = .07), but not for NF (*F* < 1). As shown in both Fig. 3 and Table 4, distances between numerical estimates based on NF were highly inaccurate, regardless of the believability of the data.

**Table 4:** Means of the distance (estimate of posterior 75 − estimate of posterior 25) and standard deviation (*SD*) for both nonnumerical and numerical estimates in Study 1

	Icon Arrays		Natural Frequencies	
	Believable	Unbelievable	Believable	Unbelievable
Nonnumerical	49.4 (29)	28.6 (37)	20.9 (27)	8.9 (35)
Numerical	39 (26)	23.4 (32)	5.3 (26)	6.1 (29)
t.test	*t*(77) = 3.16, *d* = .36***	n.s.	*t*(90) = 4.93, *d* = .52***	n.s.

***p < .001

### Comparisons between nonnumerical and numerical estimates

For NF, the percentage of nonnumerical estimates in the actual posterior ±3 interval was larger than the percentage of exact correct numerical estimates (18% vs. 9%; *McNemar* χ^2^(3)* =* 20.33*, p < .*001*,* Cohen’s* g = .*31. The opposite was true for IA (22% vs. 48%; *McNemar* χ^2^(3)* =* 45*, p < .*001*,* Cohen’s *g = .*32. The percentage of estimates in the correct direction based on NF was still significantly higher for nonnumerical rather than numerical estimates (51% vs. 39%; *McNemar* χ^2^(3) = 23.2, *p* < .001, Cohen’s *g* = .22. Nevertheless, for IA, it was similar for both estimates (74% vs. 71%). Finally, for believable scenarios, the distances between estimates (estimate of posterior 75 – estimate of posterior 25) were, on average, closer to the actual distance (50) when the estimates were reported on the line than as numerical percentages (means: 41 vs. 24 for nonnumerical vs. numerical estimates; see also Table 4).

## Discussion

Overall, results of Study 1 confirmed the observed benefit of icons, compared with text presentations, for Bayesian reasoning (e.g., Brase, 2009, 2014; Galesic et al., 2009; Garcia-Retamero & Hoffrage, 2013). Furthermore, results confirmed the hypothesis that nonnumerical estimates would foster belief-sensitive representations of the data. In each format, nonnumerical estimates were more likely in the correct direction for believable rather than for unbelievable scenarios. The believability of the data also affected the percentage of numerical estimates in the correct direction and, only for icons, the accuracy of the distance between those estimates. This supports the hypothesis that numerical estimates based on icons would be more influenced by previous beliefs than those based on text. However, accurate numerical estimates in the text format were scarce, even considering the correct direction criterion (see Table S4), suggesting a difficulty at both understanding the gist and performing the exact calculation. It might be the case that, by making the calculation easier, both the comprehension of the gist and exact calculation would be facilitated. In this case, the belief effect might be observed in the numerical estimates based on text as well. To test this hypothesis, numbers were changed in Study 2.

### Study 2

In addition to try to replicate previous findings, here we tested the hypothesis that numbers affording fraction reduction by 10 (30/40 instead of 15/20) would facilitate the calculation of the posterior 75. Such an easier calculation could also enhance comprehending the gist of the data if the numbers could be related to familiar ratios (i.e., 3/4 facilitates understanding that the posterior is larger than 1/2). If this were the case, numerical estimates would be more accurate than in Study 1, particularly for believable scenarios. We also changed “eating oranges” by “taking vitamins” as an attempt to create a preventive condition closer to the posterior 25 believable scenario. Finally, we replaced the emoticons used in the previous study by neutral circles (see an example in the Appendix) to eliminate the possibility that the format effect was due to differences in the attentional engagement to the task (Mack et al., 2002).

### Method

#### Participants and design

Two hundred sixty-five undergraduates (mean age = 22.5 years; 50 males) participated in the experiment. One hundred twenty-six received the data as ratios of natural frequencies (NF; see Appendix). The rest (139) received the same data through icon arrays (IA). In each format, some participants received believable data (59 in NF; 73 in IA) and for the rest the data were unbelievable (67 in NF; 66 in IA). Participants were randomly assigned to one of these groups.

#### Questionnaire and procedure

The questionnaire was the same as for Study 1 with a few differences. The condition “eat oranges” was replaced by “take vitamins” as a potentially stronger preventive condition. To facilitate fraction reduction, posterior 75 scenarios changed numbers (30/40 instead of 15/20). To avoid repetitions across problems, posterior 25 scenarios changed numbers as well (5/20 instead of 10/40). Finally, neutral circles instead of emoticons were used as icons (see an example in the Appendix). The rest of the procedure was as for the previous study.

#### Ethics statement

The procedure was approved by the University of Barcelona’s Bioethics Commission. Participants were free to join in the experiment and provided written consent for the use of their data for research purposes. They were debriefed in a subsequent session.

## Results

The same analyses as for the Study 1 were performed.

### Previous beliefs

Only five participants (three in IA and two in NF) showed inconsistent estimates with both believable scenarios. For belief-consistent participants, the means of these estimates were 53% for P(cold), 78% for P(cold|window open) and 38% for P(cold|orange everyday). Differences between these means were all significant (*p*s < .001).

#### Nonnumerical posterior probability estimates

**Fig. 4:**
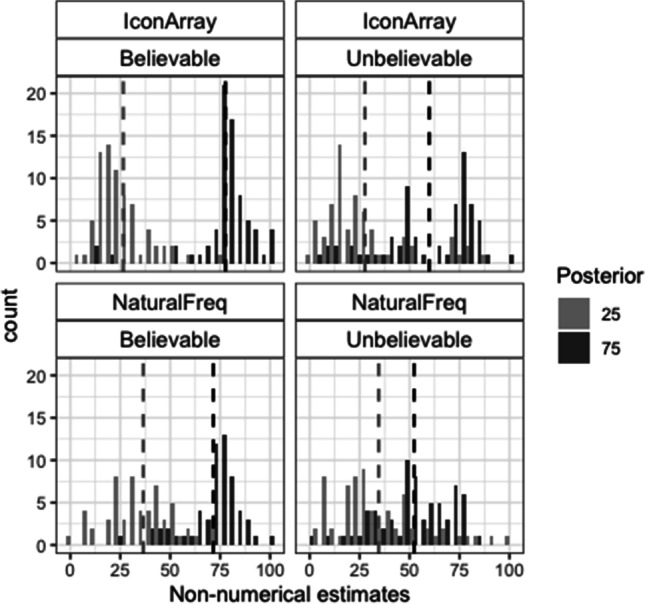
Histograms of the nonnumerical estimates in Study 2. Dashed vertical lines represent the means

Figure 4 shows the histograms of the nonnumerical estimates. The accuracy of these estimates was analyzed according to the same criteria as in the Study 1. Regarding the exact ±3 accuracy criterion, the best generalized linear mixed effects model revealed a marginal significant effect of the posterior (loglikelihood ratio with and without this factor: −288/−290; AIC ratio: 582/584; χ^2^(1) = 3.48, *p* = .064; see Table S3-A). Estimates tended to be more accurate for the posterior 75 than for the posterior 25 (28% vs. 22%; see also Table 5-A). Regarding the correct direction criterion, the model revealed significant effects of format, believability, and Posterior × Believability (loglikelihood ratio between models with and without the interactive effect: −278/−293; AIC ratio: 568/596; χ^2^(2) = 29.78, *p* < .001; see Table S3-B). The percentage of nonnumerical estimates in the correct direction was higher for IA than for NF (79% vs. 61%), and for believable rather than for unbelievable scenarios (this difference was significant only for the posterior 75: 87% vs. 48%). For believable scenarios, there were more nonnumerical estimates in the correct direction for the posterior 75 than for the posterior 25 (87% vs. 72% for posterior 75 vs. 25). For the unbelievable ones, the opposite was true (48% vs. 74% for posterior 75 vs. 25; see also Table 5-B).

**Table 5:** Percentages of correct nonnumerical estimates in Study 2 (frequencies are shown inside parentheses)

	Believable		Unbelievable	
A. Actual posterior+/-3	25	75	25	75
Icon Array	27 (20 of 73)	33 (24 of 73)	16 (10 of 63)	30 (19 of 63)
Natural Freq.	21 (12 of 57)	32 (18 of 57)	22 (15 of 67)	19 (13 of 67)
B. Correct direction	25	75	25	75
Icon Array	84 (61 of 73)	92 (67 of 73)	79 (50 of 63)	57 (36 of 63)
Natural Freq.	60 (34 of 57)	82 (47 of 57)	67 (45 of 67)	37 (25 of 67)

#### Numerical posterior probability estimates

**Fig. 5:**
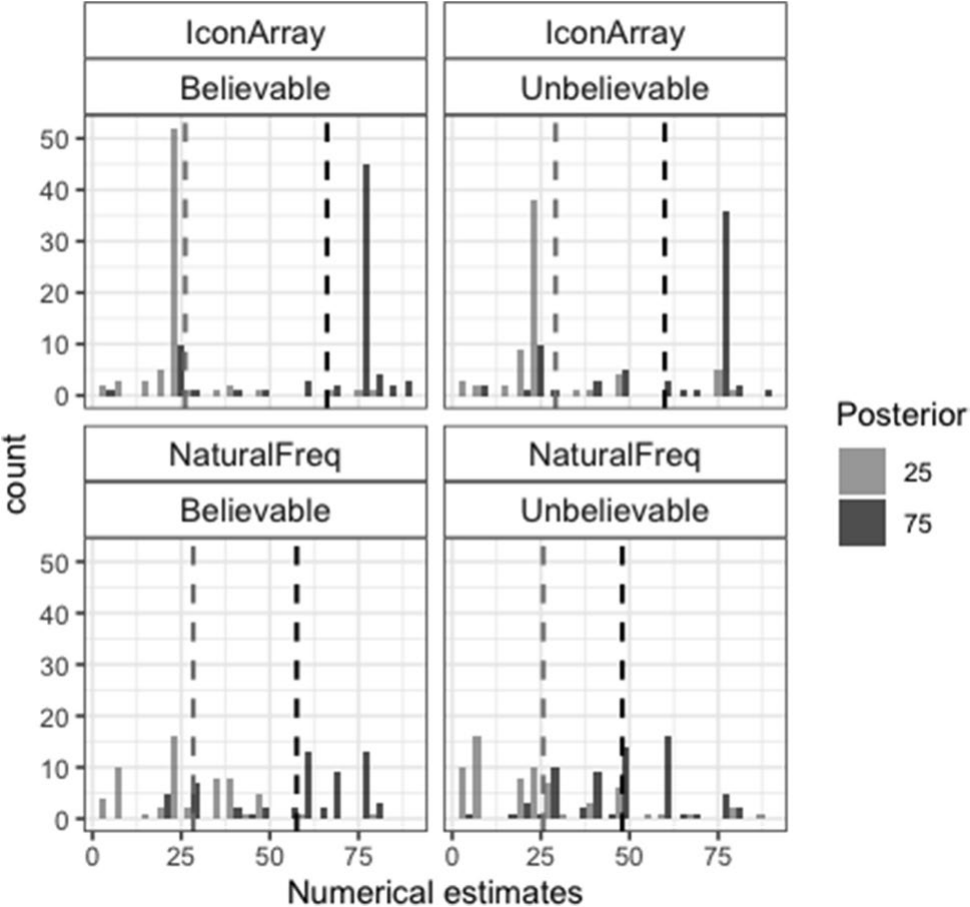
Histograms of the numerical estimates in Study 2. Dashed vertical lines represent the means

Figure 5 shows the histograms corresponding to the numerical estimates. For the exact accuracy criterion, the best model revealed significant effects of posterior, format, and believability (loglikelihood ratio with and without believability: −255/−257; AIC ratio: 520/522; χ^2^(1) = 4.89, *p* = .027; see Table S4-A). Estimates were more accurate for IA than for NF (61% vs. 18%), for believable rather than for unbelievable scenarios (47% vs. 34%), and for the posterior 25 than for the posterior 75 (44% vs. 37%; see also Table 6-A). For the correct direction criterion, in addition to the same main effects, the best model also showed significant interactions: Posterior × Format, and Posterior × Believability (loglikelihood ratio with and without interactive effects: −266/−272; AIC ratio: 547/555; χ^2^(4) = 12.13, *p* = .002; see Table S4-B). The percentage of numerical estimates in the correct direction was higher for IA than for NF (79% vs. 52%), for believable rather than for unbelievable scenarios (this difference was significant only for the posterior 75: 61% vs. 39%), and for the posterior 25 rather than for the posterior 75 (this difference was larger for NF [76% vs. 28%] than for IA [87% vs. 78%]; see also Table 6-B).

**Table 6:** Percentages of correct numerical estimates in Study 2 (frequencies are shown inside parentheses)

	Believable		Unbelievable	
A. Exact correct	25	75	25	75
Icon Array	71 (52 of 73)	62 (45 of 73)	57 (36 of 63)	54 (34 of 63)
Natural Freq.	28 (16 of 57)	23 (13 of 57)	15 (10 of 67)	7 (5 of 67)
B. Correct Direct.	25	75	25	75
Icon Array	92 (67 of 73)	77 (56 of 73)	83 (52 of 63)	62 (39 of 63)
Natural Freq.	74 (42 of 57)	46 (26 of 57)	78 (52 of 67)^a^	13 (9 of 67)

^a^Of these “correct direction” estimates, twelve were the value “10”, consequence of calculating the hit rate (“5 of 50”) for 100 (as some of the participants explained in a final questionnaire), six were the literal “5”, and seven were the value “30”, which could be derived from the false alarm rate (“15 of 50” to “30 of 100”). These superficial errors were less likely for the believable scenario of NF or for IA. Common errors for the posterior 75 estimates for NF were also the hit rate (“60 out of 100”) and the literal “30” but these did not count as correct direction estimates (see also Fig. 5).

#### Distances between estimates

The factorial ANOVAs on the distances between either the nonnumerical or numerical estimates (estimate posterior 75 − estimate posterior 25) showed significant effects of format, *F*(1, 258) = 22.78, *p* < .001, η^2^ = .08, and *F*(1, 258) = 9.51, *p* = .002, η^2^ = .04, respectively, and believability, *F*(1, 258) = 30.38, *p* < .001, η^2^ = .10, and *F*(1, 258) = 7.21, *p* = .008, η^2^ = .02, respectively, but no interaction (*F*s < 1). Distances were closer to the actual distance (50) for IA than for NF (means for nonnumerical estimates: 42 vs. 26; means for numerical estimates: 35 vs. 26), and for believable rather than unbelievable scenarios (means for nonnumerical estimates: 44 vs. 25; means for numerical estimates: 35 vs. 26; see also Table 7).

**Table 7:** Means of the distance (estimate of posterior 75 − estimate of posterior 25) and standard deviation (*SD*) in Study 2

	Icon Arrays		Natural Frequencies	
	Believable	Unbelievable	Believable	Unbelievable
Nonnumerical	51.18 (21)	31.94 (34)	35.89 (21)	17.72 (31)
Numerical	39.99 (23)	30.16 (31)	29.49 (23)	22.30 (24)
t.test	*t*(72) = 3.75***, *d* = .44	n.s.	*t*(56) = 2.31*, *d* = .31	n.s.

***p < .001, *p < .05

#### Comparisons between nonnumerical and numerical estimates

Results replicated the ones found in Study 1. For NF, the percentage of nonnumerical estimates in the posterior ±3 interval was larger than the percentage of exact correct numerical estimates (24% vs. 17%; *McNemar* χ^2^(3) = 12, *p* = .007, Cohen’s *g* = .24. For IA, the opposite was true (27% vs. 62%; *McNemar* χ^2^(3) = 54, *p* < .001, Cohen’s* g* = .38. Regarding the number of estimates in the correct direction, the difference between estimates disappeared for IA (76% for each type of estimate) but it was still significant for NF (61% vs. 50% for nonnumerical vs. numerical estimates; *McNemar* χ^2^(1) = 5.8, *p* = .02, Cohen’s* g* = .14. Finally, for believable scenarios, the distances between the estimates (estimate of posterior 75 − estimate of posterior 25) were also significantly closer to the actual distance when the estimates were provided on the line than as numerical percentages (means: 44 vs. 35; see also Table 7).

#### Comparisons between Studies 1 and 2

For IA, results of Study 2 showed more exact correct numerical estimates than in Study 1 for the posterior 75, either believable or unbelievable (overall percentages of exact correct responses were 43% vs. 58% for Study 1 vs. 2; χ^2^(1)=7.34, *p* = .007, Φ = .16), and for the believable scenario with the posterior 25 (52% vs. 71% for Study 1 vs. 2, χ^2^(1) = 5.6, *p* = .02, Φ = .19). For NF, differences between studies were significant only for the believable scenarios, either with the posterior 75 (10% vs. 22% for Study 1 vs. 2; χ^2^(1) = 4.33, *p* = .04, Φ = .17) or with the posterior 25 (9% vs. 27% for Study 1 vs. 2; χ^2^(1) = 7.8, *p* = .005, Φ = .23).

## Discussion

Results of Study 2 replicated the main findings of Study 1. Iconic presentations and believable data produced more accurate posterior probability estimates than textual presentations and unbelievable data. Interestingly, regarding the likelihood of nonnumerical estimates in the correct direction, the effect of the posterior reversed in believable scenarios (more accurate estimates for the posterior 75 than for the posterior 25). This, together with the main belief effect on the accuracy of the numerical estimates, confirms that numbers pointing to familiar ratios such as 3/4 enhanced gist, belief-sensitive comprehension. Nevertheless, numerical estimates were still less accurate for the posterior 75 than for the posterior 25, particularly for the text format. Of note, the numerator of the hit rate described in the posterior 75 scenarios (“30”) was still relatively small, which could explain the bias towards lower estimates. Indeed, this value was one of the frequent responses for the posterior 75 in NF (see Fig. 5). In sum, compared with Study 1, estimates were in general more accurate in the present study, but such improvement seemed to depend on the format and on the believability of the data.

### Study 3

Studies 1 and 2 consistently showed that the belief effect was stronger for nonnumerical rather than numerical probability estimates. Still, the belief effect was significant on the percentage of numerical estimates in the correct direction, particularly when the data were presented through icons (distances between estimates were closer to the actual distance). Given that both numerical and nonnumerical requests required to infer a single-event probability, it might be the case that participants answered both based on the gist of the posterior ratio, producing such belief-sensitive estimates. That is, the request of either a single mark on a line or a single percentage may have promoted the integration of the different pieces of numerical information, facilitating a gist, belief-sensitive understanding of the data. In contrast, numerical estimates in frequency format (e.g., “among the people sleeping with the window open, how many catch a cold? __ of __”; see also the Appendix) might promote focusing more on the critical numbers (“people sleeping with the window open” and, among them, “people catching a cold”), rather than on the ratio. In this case, participants would mostly rely on verbatim exact processing and, therefore, a much weaker belief effect on the accuracy of the numerical estimates would be expected. The present study aimed to test this hypothesis. As previously shown for NF formats (e.g., Cosmides & Tooby, 1996), we also expected more accurate numerical estimates for frequency rather than single-event probability requests of the posterior ratio.

### Method

#### Participants and design

One hundred ninety-one undergraduates (mean age = 21.8 years; 46 males) participated in the experiment. Ninety-five received the data as ratios of natural frequencies (NF; see Appendix). The rest (96) received the same data through icon arrays (IA). In each format, some participants received believable data (49 in NF; 48 in IA) and for the rest the data were unbelievable (46 in NF; 48 in IA). Participants were randomly assigned to one of these groups.

#### Questionnaire and procedure

The questionnaire was the same as for Study 2, except for the request of the numerical posterior probability. After the nonnumerical probability request, the following question was included: “More concretely, among the people who /sleep with the window open/take vitamins/, how many will catch a cold?” (_ of _ ; see also the Appendix). The rest of the procedure was as for the previous studies.

#### Ethics statement

The procedure was approved by the University of Barcelona’s Bioethics Commission. Participants were free to join in the experiment and provided written consent for the use of their data for research purposes. They were debriefed in a subsequent session.

### Results

The same analyses as for the previous studies were performed.

#### Previous beliefs

Only six participants (three in each format) showed inconsistent estimates with both believable scenarios. For belief-consistent participants, the means of these estimates were 67% for P(cold), 87% for P(cold|window open) and 45% for P(cold|orange everyday). Differences between these means were all significant (*p*s < .001).

#### Nonnumerical posterior probability estimates

**Fig. 6:**
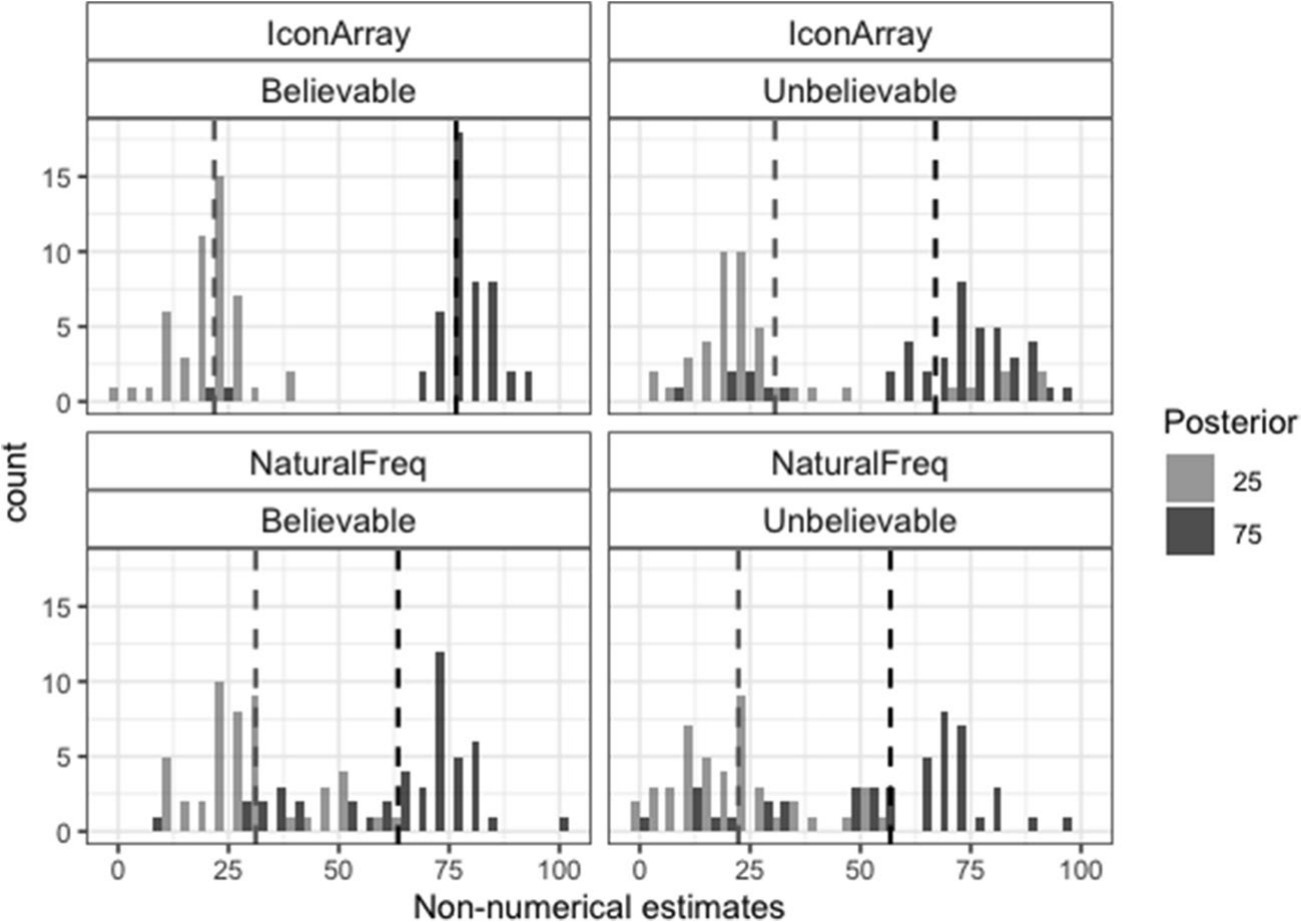
Histograms of the nonnumerical estimates in Study 3. Dashed vertical lines represent the means

Figure 6 shows the histograms of the nonnumerical estimates. The accuracy of these estimates was analyzed according to the same criteria as in the previous studies. Regarding the actual posterior ±3 accuracy criterion, the best generalized linear mixed-effects model revealed significant effects of format and believability (loglikelihood ratio with and without believability: −223/−227; AIC ratio: 453/469; χ^2^(1) = 8.07, *p* = .005; see Table S5-A). Estimates based on IA were more accurate than those based on NF (36% vs. 26%). Estimates were also more accurate for believable rather than unbelievable scenarios (40% vs. 26%; see also Table 8-A). Regarding the correct direction criterion, the model revealed significant effects of posterior, format, and believability (loglikelihood ratio between models with and without believability: −158/−161; AIC ratio: 325/330; χ^2^(2) = 6.16, *p* = .013; see Table S5-B). The percentage of nonnumerical estimates in the correct direction was higher for IA than for NF (90% vs. 74%), for believable rather than for unbelievable scenarios (86% vs. 77%), and for the posterior 25 rather than the posterior 75 (87% vs. 76%; see Table 8-B).

**Table 8:** Percentages of correct nonnumerical estimates in Study 3 (frequencies are shown inside parentheses)

	Believable		Unbelievable	
A. Actual posterior +/-3	25	75	25	75
Icon Array	50 (24 of 48)	48 (23 of 48)	31 (14 of 45)	27 (12 of 45)
Natural Freq.	34 (16 of 47)	26 (12 of 47)	27 (12 of 45)	16 (7 of 45)
B. Correct Direct.	25	75	25	75
Icon Array	100 (48 of 48)	96 (46 of 48)	84 (38 of 45)	78 (35 of 45)
Natural Freq.	77 (36 of 47)	72 (34 of 47)	87 (39 of 45)	58 (26 of 45)

#### Numerical posterior probability estimates

**Fig. 7:**
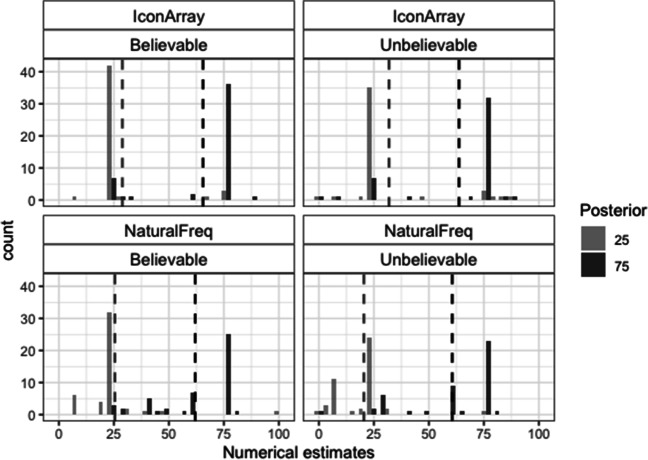
Histograms of the numerical estimates in Study 3. Dashed vertical lines represent the means

Figure 7 shows the histograms corresponding to the numerical estimates. For the exact accuracy criterion, the best model revealed significant effects of posterior and format (loglikelihood ratio with and without format: −205/−213; AIC ratio: 419/432; χ^2^(1) = 15.17, *p* < .001; see Table S6-A). Estimates were more accurate for IA than for NF (78% vs. 56%), and for the posterior 25 than for the posterior 75 (72% vs. 63%). Responses tended to be also more accurate for believable rather than for unbelievable scenarios, but the difference was not significant (see Table 9-A). For the correct direction criterion, in addition to the same main effects, the best model also showed significant Posterior × Format (loglikelihood ratio with and without the interactive effect: −163/−168; AIC ratio: 336/345; χ^2^(1) = 10.62, *p* = .001; see Table S6-B). The percentage of numerical estimates in the correct direction was higher for the posterior 25 rather than for the posterior 75 (87% vs. 67%). For the posterior 25, the accuracy of the estimates did not differ between formats, but they did for the posterior 75 (percentage of estimates in the correct direction were 78% and 56% for IA and NF, respectively; see also Table 9-B).

**Table 9:** Percentages of correct numerical estimates in Study 3 (frequencies are shown inside parentheses)

	Believable		Unbelievable	
A. Exact Correct	25	75	25	75
Icon Array	88 (42 of 48)	75 (36 of 48)	78 (35 of 45)	71 (32 of 45)
Natural Freq.	68 (32 of 47)	53 (25 of 47)	53 (24 of 45)	51 (23 of 45)
B. Correct Direct.	25	75	25	75
Icon Array	92 (44 of 48)	77 (37 of 48)	84 (38 of 45)	78 (35 of 45)
Natural Freq.	94 (44 of 47)	55 (26 of 47)	98 (44 of 45)^a^	56 (25 of 45)

^a^Of these “correct direction” estimates, twelve were the value “10”, consequence of calculating the hit rate (“5 of 50”) for 100 (as some of the participants explained in a final questionnaire; see also Fig. 7)

#### Distances between estimates

The factorial ANOVA on the distances between the nonnumerical estimates (estimate posterior 75 − estimate posterior 25) showed significant effects of format, *F*(1, 181) = 11.74, *p* < .001, η^2^ = .06, believability, *F*(1, 181) = 4.99, *p* = .07, η^2^ = .03, and a significant interaction, *F*(1, 181) = 7.93, *p* = .005, η^2^ = .04. As shown in Table 10, the effect of format was significant only for believable scenarios, and the effect of believability was significant only for icons. For numerical estimates, the ANOVA on the distances reported no significant effect.

**Table 10:** Means of the distance (estimate of posterior 75 − estimate of posterior 25) and standard deviation (*SD*) in Study 3

	Icon Arrays		Natural Frequencies	
	Believable	Unbelievable	Believable	Unbelievable
Nonnumerical	54.83 (16)	36.38 (34)	32.28 (24)	34.47 (23)
Numerical	36.65 (22)	31.82 (31)	36.51 (21)	40.16 (17)
t.test	*t*(43) = 5.43 ***, *d* = .78	n.s.	n.s.	n.s.

***p < .001

#### Comparisons between nonnumerical and numerical estimates

In both formats, the percentage of exact correct numerical estimates was larger than the percentage of nonnumerical estimates in the posterior +/-3 interval (IA: 78% vs. 39%; *McNemar* χ^2^(3) = 43.23, *p*<.001, *Cohen’s g*=.41; NF: 57% vs. 26%; *McNemar* χ^2^(3) = 35.44, *p* < .001, Cohen’s* g* = .39). Regarding the percentage of estimates in the correct direction, the difference between estimates was also significant, although in this case, percentages were larger for nonnumerical rather than numerical estimates: IA: 83% vs. 79%; *McNemar* χ^2^(3) = 9.2, *p* = .026, Cohen’s* g* = .32; NF: 76% vs. 73%; *McNemar* χ^2^(3) = 7.8, *p* = .050, Cohen’s* g* = .16). Finally, only for the believable scenarios in iconic format, the distance between the estimates (estimate of posterior 75 − estimate of posterior 25) was significantly closer to the actual distance when the estimates were provided on the line than as numerical percentages (means: 55 vs. 37; see Table 10).

## Discussion

As expected, numerical estimates of the posterior ratio were more accurate when requested as frequencies in set and subset (Study 3) than as single-event probabilities (Studies 1 and 2). Differing from the previous studies, numerical estimates in either format were more accurate than the nonnumerical ones when the exact accuracy criterion was used. This finding confirms that the frequency question promoted exact verbatim processing, regardless the believability of the data. However, for both formats, nonnumerical estimates were more likely in the correct direction than the numerical ones (for NF, this was the case in the three studies). That is, nonnumerical single-event probability requests seemed to have facilitated understanding the gist of the posterior ratio to a larger extent than the frequency questions. In line with this proposal, and as observed in Studies 1 and 2, nonnumerical estimates were more accurate for believable rather than unbelievable scenarios.

### General discussion

The present research aimed to test the hypothesis that the influence of previous beliefs on Bayesian reasoning would mainly be observed in conditions fostering a gist comprehension of the data. Accordingly, we expected to observe stronger belief effect for iconic rather than for textual presentations, and for nonnumerical rather than for numerical estimates. Here, we discuss the results concerning each of these predictions.

### Presentation format and gist processing

Overall, results replicated the observed benefit of icons for Bayesian reasoning (e.g., Brase, 2009, 2014; Galesic et al., 2009; Garcia-Retamero et al., 2015; Tubau et al., 2019). In the three studies, icons promoted more accurate estimates (either expressed nonnumerically or numerically) than natural frequencies in text format. Moreover, estimates based on icons were, overall, more affected by previous beliefs, being the distance between estimates closer to the actual distance (50) in believable scenarios. These findings are coherent with the role of icon arrays in helping to grasp the gist of the represented ratios (Brust-Renck et al., 2013; Stone et al., 2018; Tubau et al., 2019) and, as shown in the present studies, in promoting belief-sensitive representations. Importantly, the benefit of icons for Bayesian reasoning might be due to the specific organization used in the present experiments, where the requested reference class is inside the frame (see Fig. 1). It has been shown that other organizations of icons are less effective (e.g., Witt & Dhami, 2022).

By contrast, replicating previous findings with probability or proportion requests (Cosmides & Tooby, 1996; Tubau et al., 2019; Weber et al., 2018), natural frequencies described in the text produced mostly inaccurate numerical percentages, supporting a more superficial processing of the data (further discussion on the limitations of textual presentations for Bayesian reasoning can be read in Tubau, 2022). In Study 2, where the numbers facilitated the comprehension of the gist, the numerical estimates improved for the believable scenarios based on natural frequencies described in the text. Accordingly, although the accuracy of the numerical estimates was still low, these results suggest that Bayesian inferences based on numerical descriptions may also benefit from conditions fostering a gist understanding. Differing from single-event probability requests, posterior probabilities requested as frequencies (Study 3) promoted verbatim exact processing, increasing the number of correct estimates, and reducing the influence of previous beliefs in both formats.

Of note, comparisons between the Studies 1 and 2 suggest that, for the iconic presentation, numbers affording easier calculation (i.e., “30 of 40” instead of “15 of 20”) might have increased the likelihood of getting the correct response through either verbatim calculation or gist-based reasoning. This could explain the general increment in Study 2 of correct numerical responses in the iconic format^3^. However, differing from the iconic format, the accuracy of the numerical estimates based on the frequencies included in the text only improved for believable scenarios. This, together with the finding that exact correct numerical estimates were still scarce, suggests that numbers included in Study 2 facilitated the comprehension of the gist rather than exact calculation. Nevertheless, further research is needed to investigate the specific impact of different Bayesian reasoning strategies and their relationship with the complexity of the calculation and the presentation format.

Results of Study 2 also suggest that unbelievable data promoted superficial processing of the data rather than an adjustment of the estimate based on previous beliefs. This was the case particularly for the text presentation, where the most frequent erroneous responses were the hit rate and the base rate (see Fig. 5). This appears to be in contrast with the findings of Cohen et al. (2017), where unbelievable data seemed to promote an adjustment of the estimates (estimates between the actual posteriors and the previous beliefs). However, it is unknown the extent to which some of the erroneous responses in Cohen et al. (2017) corresponded to superficial errors as well. That is, superficial errors in unbelievable scenarios (e.g., selecting the hit rate or the base rate alone) could resemble responses towards the previous belief (incorrect direction), as found in the present studies for the posterior 75 (see Figs. 3 and 5). It might also be the case that unbelievable data promote more superficial reasoning in more difficult tasks (exact instead of approximate estimate as provided by the participants of Cohen et al.).

For the iconic format, results also suggest that unbelievable data promoted a change in the strategy, but different from the text format (many of the errors seemed to be consequence of inverting the colors of the icon array). Overall, these findings are coherent with an initial evaluation of the consistency or believability of the gist of the posterior. This process might be faster in conditions such as iconic presentations or, as found in other reasoning tasks, for more skilled participants (e.g., Bago & De Neys, 2019; Furlan et al., 2016; Raoelison et al., 2020). In the case of our problems, when the gist was believable, participants proceeded with the calculation. But if it was evaluated as unbelievable, some of them seemed to apply a different strategy. Future research could investigate these suggestions in more detail.

### The role of nonnumerical probability estimates

Based on the proposals that nonnumerical estimates foster intuitive or gist comprehension (Dixon & Moore, 1996; Mathews & Ellis, 2018), we expected that estimates on the line would be particularly affected by the believability of the data. Supporting this expectation, the percentage of nonnumerical estimates in the correct direction were, in each presentation format, higher for believable rather than for unbelievable scenarios. However, a weaker but significant belief effect was observed for the accuracy of numerical estimates expressed as single-event probabilities as well, and this effect was modulated by format in Study 1 (the numerical estimates of the posterior 75 were highly inaccurate for NF, regardless of the believability of the data). Cohen et al. (2017) also reported a significant belief effect for Bayesian problems in a text format, but the response choices (lines of radio buttons in 5 % increments) were closer to the present nonnumerical rather than numerical requests. The fact that in the present experiments the numerical estimates were reported after the nonnumerical ones might explain the general belief effect in either format. Also, as Study 2 suggested, the complexity of the calculation may also determine the extent to which the gist would be understood and, therefore, probability estimates would be affected by previous beliefs.

Importantly, these results confirmed the claim that nonnumerical representations of proportions may be, in some conditions, more accurate than corresponding numerical expressions (Ahl et al., 1992; Dixon & More, 1996; but see below). This was the case for the textual presentations of Studies 1 and 2, even when considering the estimates in the correct direction. That is, participants reading the natural frequencies demonstrated better gist understanding of the posterior probability when nonnumerical rather than numerical single-event probability estimates were requested. This supports the hypothesis that requests of nonnumerical estimates reduce the tendency to rely on superficial-verbatim processing of the text, compared with the requests of numerical ones. At the same time, by fostering a gist comprehension of the posterior probability, nonnumerical estimates might have helped to understand the impact of the condition, which has been also related to a more intuitive processing of the data (Tentori et al., 2016).

In Study 3, the advantage of nonnumerical estimates over the numerical ones was true only regarding the correct direction criterion. Given that the frequency questions more explicitly referred to the numbers that had to be selected, they promoted a more verbatim exact processing, increasing the number of exact correct responses. This is in line with findings using other tasks (e.g., Tentori et al., 2016) where numerical estimates tend to be more accurate than the nonnumerical ones. Accordingly, the benefit of nonnumerical estimates over the numerical ones seems to depend on the format of the data (iconic or text), the type of response (single-event probability or frequency format), and on the accuracy criterion (exact or correct direction).

For the iconic format of the three studies, there were more exact correct numerical estimates than nonnumerical estimates in the actual posterior ±3 interval. However, the gist comprehension of the iconic data seemed to be independent from the question when requested as a single-event probability, as shown by the similar percentage of numerical and nonnumerical estimates in the correct direction in Studies 1 and 2 (as commented above, in Study 3, nonnumerical single-event probability estimates were more likely in the correct direction than the estimates expressed as frequencies in set and subset). This suggests that, as observed with the gist perception of visual images (e.g., Furtak et al., 2022), the perception of the ratio of colors occurred very fast, before the processing of the requests. Nevertheless, for the believable scenarios, the distance between the posterior estimates based on icons was, in the three studies, closer to the actual distance when the estimates were expressed nonnumerically rather than numerically. Therefore, nonnumerical probability requests could be helpful to improve the comprehension of the gist of the posterior probability in both text and iconic presentations.

## Concluding remarks

Present findings support the proposal that iconic presentations promote a gist, belief-sensitive understanding of the quantitative relations. In three studies, Bayesian inferences were more accurate, and in some conditions more affected by the believability of the data, when the numerical information could be visualized in arrays of icons rather than inferred from the text. Of note, this benefit was observed for posterior probability estimates expressed either as single-event probabilities, or as frequencies of individuals. Therefore, icons seem to specifically facilitate the comprehension of the posterior ratio beyond the counting of frequencies in set and subset. For text presentations, requesting nonnumerical estimates seemed also to enhance the comprehension of the gist of the data, reducing the tendency to rely on superficial reasoning, particularly in the case of believable data. Although gist-based reasoning may have some costs due to stronger affective reactions than verbatim processing, gist comprehension may be necessary to detect belief-data inconsistencies, being a first step for changing previous beliefs if it were required. In this regard, promoting gist comprehension of the numerical data might be particularly critical when misinformation, prejudices, or wrong beliefs should be overcome.

### Supplementary Information

Below is the link to the electronic supplementary material.

Supplementary file1 (DOCX 27 KB)

## Appendix

Note: Here is an example of the icon array used in Studies 2 and 3. An example of the icon array used in Study 1 can be seen in Fig. 1 of the main text.

The following figure shows data from a recent study about the number of people who catch a cold in winter and the number of people who sleep with the window open.

Nonnumerical posterior probability request (in the three studies, both formats)

*Based on these data, to what extent will a person who sleeps with the window open catch a cold?*

Numerical posterior probability request (Studies 1 and 2, both formats)

*More concretely, according to the data presented above, what is the probability that a person who sleeps with the window open will actually catch a cold? (%)*

Numerical posterior probability request (Study 3, both formats)

*More concretely, according to the data presented above, among the people who sleep with the window open, how many will actually catch a cold? (“ _of__”)*

## Footnotes

1 In Tubau et al. (2019) the single-event probability was requested as two terms (i.e., “based on previous data, what is the probability that a person with the condition is actually ill: _ of _”), requiring a single addition as for the typical frequency question (“of the individuals with the condition, how many are ill? _ of _”). Based on the data in Fig. 1, the response should be “15 of [15+5]” (or equivalent expression) in both cases.

2 For example, in Table 1 (posterior 75), the numbers quantifying the subsets (15 and 5) are aligned with “the probability that an individual sleep with the window open” (probability of the datum) because they can keep the subset role ([15+5] of 100). By contrast, these numbers are misaligned with “the probability that an individual who sleeps with the window open will catch a cold” (posterior probability) because they must change the relational role from subset to reference class (15 of [15+5]; see also Johnson & Tubau, 2017; Tubau, 2022).

3 Study 2 also showed more exact correct numerical responses than Study 1 for the believable scenario with posterior 25 in each format. However, rather than due to easier calculation, this improvement might be related with the ratio bias phenomenon (Denes-Raj et al., 1995; Reyna & Brainerd, 2008). That is, smaller numerators (i.e., 5/20 vs. 10/40), together with believable data, might have helped to intuitively understand that the probability was low.
