# Supplementary material for: Previous beliefs affect Bayesian reasoning in conditions fostering gist comprehension
Source: Mem Cognit. 2023 Jun 2;51(8):1819–35. doi: 10.3758/s13421-023-01435-1 (PMC10638198; doi:10.3758/s13421-023-01435-1)
Supplement: Supplementary file 1 — Supplementary file1 (DOCX 27 KB) [file 13421_2023_1435_MOESM1_ESM.docx]

**Previous beliefs affect Bayesian reasoning in conditions fostering gist comprehension**

Running Head: Gist comprehension in Bayesian reasoning

Elisabet Tubau*, Àngels Colomé, Javier Rodríguez-Ferreiro

Departament de Cognició, Desenvolupament i Psicologia de l’Educació

Institut de Neurosciències

Universitat de Barcelona

**Supplementary Data**

*Corresponding author

Elisabet Tubau

Departament de Cognició, Desenvolupament i Psicologia de l’Educació

(secció Processos Cognitius)

Pg Vall d’Hebron, 171

O8035 Barcelona (Spain)

[etubau@ub.edu](mailto:etubau@ub.edu)

**Supplementary Data - Study1**

Table S1. Results of the Deviance analyses of the binomial generalized linear mixed effects models (Type II Wald chi-square tests) for non-numerical estimates’ accuracy in Study 1

| 1. Accuracy criterion: actual posterior +/-3 | | | 1. Accuracy criterion: correct direction | | |
| --- | --- | --- | --- | --- | --- |
| Factor | χ^2^ (df) | p | Factor | χ^2^ (df) | p |
| Posterior (P) | 5.91 (1) | .015 | Posterior (P) | 26.18 (1) | <.001 |
|  | | | Format (F) | 26.60 (1) | <.001 |
|  |  |  | Believability (B) | 13.29 (1) | <.001 |
|  |  |  | PxF | 4.80 (1) | .028 |
|  |  |  | PxB | 4.37 (1) | .037 |

Table S2. Results of the Deviance analyses corresponding to the binomial generalized linear mixed effects model (Type II Wald chi-square tests) for the accuracy of numerical estimates in Study 1

| 1. Acuracy criterion: exact correct response | | | 1. Acuracy criterion: correct direction | | |
| --- | --- | --- | --- | --- | --- |
| Factor | χ^2^ (df) | p | Factor | χ^2^ (df) | p |
| Posterior (P) | 5.90 (1) | .012 | Posterior (P) | 57.57(1) | <.001 |
| Format (F) | 48.23(1) | <.001 | Format (F) | 52.81(1) | <.001 |
| PxF | 3.31 (1) | .069 | Believability (B) | 6.91(1) | .008 |
|  | | | PxF | 6.84(1) | .008 |

**Supplementary Data - Study 2**

Table S3. Results of the Deviance analyses corresponding to the binomial generalized linear mixed effects model (Type II Wald chi-square tests) for the accuracy of non-numerical estimates in Study 2.

| 1. Acc criterion: actual posterior +/-3 | | | 1. Acc criterion: correct direction | | |
| --- | --- | --- | --- | --- | --- |
| Factor | χ^2^ (df) | P | Factor | χ^2^ (df) | p |
| Posterior (P) | 3.41 (1) | .064 | Posterior (P) | 1.56 (1) | .212 |
|  | | | Format (F) | 14.81 (1) | <.001 |
|  |  |  | Believability (B) | 10.52(1) | .001 |
|  |  |  | PxB | 29.95 (1) | <.001 |

Table S4. Results of the Deviance analyses corresponding the binomial generalized linear mixed effects model (Type II Wald chi-square tests) for the accuracy of numerical estimates in Study 2

| 1. Acc criterion: exact correct response | | | 1. Acc criterion: correct direction | | |
| --- | --- | --- | --- | --- | --- |
| Factor | χ^2^ (df) | P | Factor | χ^2^ (df) | p |
| Posterior (P) | 6.39 (1) | .011 | Posterior (P) | 34.01 (1) | <.001 |
| Format (F) | 16.32 (1) | <.001 | Format (F) | 23.62 (1) | <.001 |
| Believability (B) | 6.46 (1) | .011 | Believability (B) | 7.26 (1) | .007 |
|  | | | PxF | 5.39 (1) | .021 |
|  |  |  | PxB | 5.28 (1) | .021 |

**Supplementary Data - Study 3**

Table S5. Results of the Deviance analyses corresponding to the binomial generalized linear mixed effects model (Type II Wald chi-square tests) for the accuracy of non-numerical estimates in Study 3.

| 1. Acc criterion: actual posterior +/-3 | | | 1. Acc criterion: correct direction | | |
| --- | --- | --- | --- | --- | --- |
| Factor | χ^2^ (df) | p | Factor | χ^2^ (df) | p |
| Format | 6.85 (1) | .009 | Format | 12.26 (1) | <.001 |
| Believability | 7.59 (1) | .006 | Believability | 5.39 (1) | .020 |
|  | | | Posterior | 8.41 (1) | .004 |

Table S6. Results of the Deviance analyses corresponding the binomial generalized linear mixed effects model (Type II Wald chi-square tests) for the accuracy of numerical estimates in Study 3

| 1. Acc criterion: exact correct response | | | 1. Acc criterion: correct direction | | |
| --- | --- | --- | --- | --- | --- |
| Factor | χ^2^ (df) | p | Factor | χ^2^ (df) | p |
| Posterior (P) | 6.26 (1) | .011 | Posterior (P) | 18.57 (1) | <.001 |
| Format (F) | 10.40 (1) | .001 | Format (F) | 2.71 (1) | .099 |
|  | | | PxF | 9.26 (1) | .002 |
